# Influence of harvest stage on the pharmacological effect of *Angelica dahurica*

**DOI:** 10.1186/s40529-018-0230-1

**Published:** 2018-05-15

**Authors:** Wei-Hong Liang, Tung-Wu Chang, Yuh-Chyang Charng

**Affiliations:** 1 Department of Agronomy, National Taiwan University, Taipei, Taiwan Republic of China; 2 Hualien District Agricultural Research and Extension Station, Council of Agriculture, Executive Yuan, Hualien, Taiwan Republic of China

**Keywords:** *Angelica dahurica*, Summer dormancy, Harvest time, Furanocoumarin, Antioxidant

## Abstract

**Background:**

Baizhi (*Angelica dahurica*) has been widely used as a traditional Chinese herbal medicine, functional food and cosmetic product ingredient, mostly because of the high furanocoumarin compounds in roots. The cropping system of Baizhi with its unique summer dormancy feature, is easily affected by the transition of its growth stages. The aim of this study was to analyze the quantity (size, form and dry weight [DW]) and quality (antioxidant and furanocoumarin content) of taproot and lateral root from three growth stages of Baizhi; vegetative (V-stage), summer dormancy (S-stage) and bolting stage (B-stage).

**Results:**

Root length and diameter were lower at V-stage than the other two stages, and S-stage had higher lateral root to total root ratio. However, the highest root DW was observed at S-stage. Antioxidant activity was revealed by 2,2-diphenyl-l-picrylhydrazyl and Fe^2+^ chelating assay, and the content of six furanocoumarin compounds, including xanthotoxin, bergapten, oxypeucedanin, imperatorin, phellopterin and isoimperatorin, was analyzed by liquid chromatography. Although the antioxidant activity was less at S-stage than the other stages, furanocoumarin contents showed little variation.

**Conclusion:**

Considering the high DW and stable furanocoumarin composition, S-stage is the best harvest stage than the other stages because of its richer total pharmacological content.

## Background

Baizhi (*Angelica dahurica* BENTH. et HOOK.) is a perennial Apiaceae plant, originated in Taiwan (Wang et al. 2001) and found abundantly in Korea, China, Japan and Russia (Sarker and Nahar 2004; Li et al. 2014a, b). The root of Baizhi is a traditional Chinese medicine known for its antipyretic and analgesic properties since thousands of years ago. Today, researchers are analyzing the multiple biological functions and investigating the chemical constituents of Baizhi.

The multiple pharmacological effects of Baizhi root extracts include antioxidation (Piao et al. 2004), anti-inflammatory (Pervin et al. 2014), antiproliferative (Yousif et al. 1999), skin whitening (Cho et al. 2006), anti-tumor (Kim et al. 2007), antimicrobial (Kwon et al. 1997) and anti-Alzheimer effects (Marumoto and Miyazawa 2010). These effects are believed to be attributed to the plant’s rich furanocoumarin compounds such as imperatorin and isoimperatorin. Imperatorin has anti-inflammatory (Garcia et al. 2000), anticonvulsant (Luszczki et al. 2007), hepatoprotective (Oh et al. 2002), myorelaxant (Chiou et al. 2001), vasodilator (Wang et al. 2016) and anti-cancer effects (Li et al. 2014a, b). Isoimperatorin has anti-inflammatory (Abad et al. 2001), antiallergic (Ryu et al. 2001) and antimicrobial effects (Suleimenov 2009). Beside the pharmacological effects of Baizhi furanocoumarins, root extracts contain a variety of phenolic compounds that are connected to its strong antioxidant activity (Wang et al. 2017). Recently, foods with antioxidant effects become valued for their ability to remove reactive oxygen species, which oxidize lipids, DNA, membranes and proteins, and are involved in atherosclerosis, cancers and other diseases (Leopold and Loscalzo 2009).

Growth of Baizhi has three stages: V-stage, vegetative stage; B-stage, bolting stage; and S-stage, summer dormancy stage. Summer dormancy in herbaceous perennials is characterized by: (1) cessation or reduction of leaf meristem growth; (2) senescence of most or all above-ground herbage; (3) possible dehydration of the bases of the youngest leaves at the base of vegetative tillers; and (4) possible preceding formation of resting organs in the form of swollen basal internodes (corm/tuber) or swollen leaf bases (such as bulbs) (Volaire and Norton 2006). Although a report indicated NtFTs are target genes of temperature signal pathway to regulate the dormancy process in *Narcissus tazetta* var *Chinensis* (Feng et al. 2015), the relationship between biochemical regular and summer dormancy still lacked conclusive researches (Gillespie and Volaire 2017). For environmental drivers, vegetative organs develop under increasing day-length and temperature at the end of spring will induce summer dormancy (Volaire et al. 2009). In addition, water deficit was shown to be an induction factor for *P. bulbosa* L. (Ofir and Kigel 2007) and *D. glomerata* (Volaire 2002). Accordingly, after the V-stage, instead of transitioning to the B-stage, Baizhi enters summer dormancy (S-stage) to survive hot and dry environmental conditions. Its above-ground portions die during summer, and its root becomes the storage organ and therefore becomes heavier, a mechanism that implies more harvest yield than at other growth stages. Thus, it is expected that Baizhi roots should be harvested at S-stage, although the S-stage is not necessary to complete the regular life cycle of Baizhi.

A previous study arranged a series of sowing dates, from September to November, to analyze the B-/S-stage ratio in Baizhi (Xingfu et al. 1999). However, few analyses have revealed the content of pharmacological compounds in Baizhi roots extracted from different stages. Accordingly, the quantities in root may change during the growth stages. For example, with *Angelica sinensis* (known as Danggui in China), a well-known functional food for its roots, the growth stage is the most important factor affecting functional compound composition (Lü et al. 2009). Another study indicated that the content of six phthalides and four aromatic acids in *A. sinensis* varied with harvest timing (Qian et al. 2013).

In this study, we aimed to determine the functional activities of Baizhi roots at different growth stages, specifically the S-stage, which rarely occurs in other root-based medicinal plant species. To this end, we determined the activity of six furanocoumarin compounds, xanthotoxin, bergapten, oxypeucedanin, imperatorin, phellopterin and isoimperatorin, and antioxidants in Baizhi primary taproots and lateral roots at V-, S- and B-stages. The results will provide experimental proof for the harvest timing of Baizhi, especially for functional quality.

## Materials and methods

### Plant materials

**Fig. 1:**
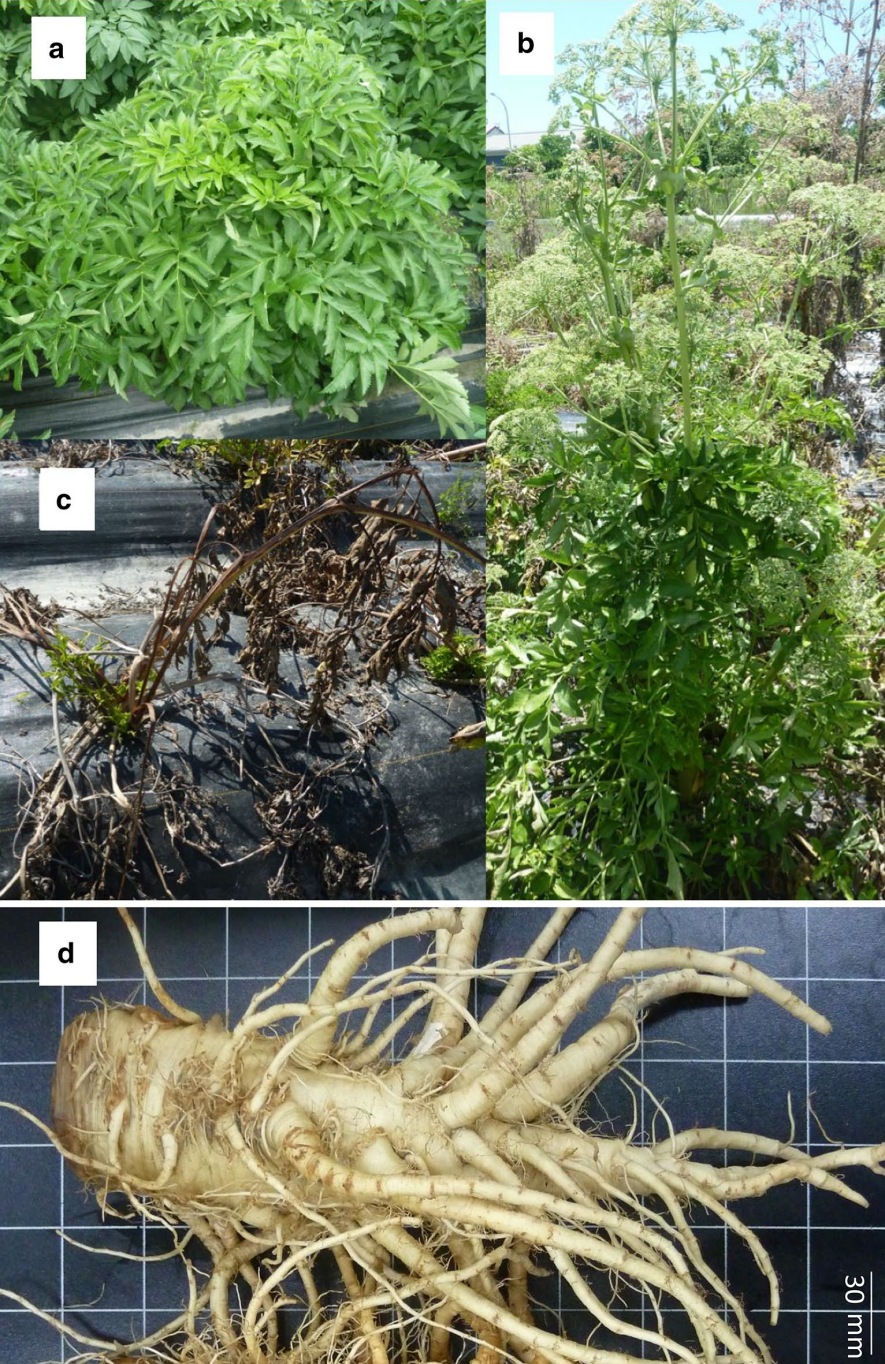
The appearance of Baizhi at three growth stages: V-stage, vegetative (23 weeks after sowing, **a**); B-stage, bolting (34 weeks after sowing, **b**); S-stage, summer dormancy (38 weeks after sowing, **c**); and roots harvested at V-stage (**d**)

The Baizhi field experiment of our study was grown in year 2014–2015 on a sandy loam soil at the Hualien District Agricultural Research and Extension Station (121°33E, 23°58N), Council of Agriculture, Executive Yuan. The 30 main plots 10 × 1.2 × 0.3 m (L × W × H) were sown in October and November 2014. Seed was initially sown to dry seedbeds and transplanted as one plant per hill to the puddled field plots at 3–4 leaves stage. The site soils had pretreated (2 weeks before transplantation) with organic matter (N:P_2_O_5_:K_2_O = 4.9:2.1:1.9; 8000 kg ha^−1^). The experiment was conducted one plant per hill to the puddled field with regular water application. Hills were spaced at a distance of 120 cm with 60 cm between planted rows (hill spacing = 120 cm × 60 cm). During the culture period, the total rainfall is about 900 mm, the mean air temperatures range from about 17 to 29 °C, the mean monthly sunshine hours range from 70 to 230 h. It was harvested at the three harvest stages, vegetative (V-stage), bolting (B-stage) and summer dormancy (S-stage), during the following year in May, July and August based on plant appearance (Fig. 1). Ten plantlets were harvested at each stage. The taproots and lateral roots (Fig. 1d) were separated, and the length, diameter and fresh weight were measured. The roots were then sliced, and part of the fresh root slices were freeze-dried (LYPH. LOCK 18, Labconco, USA) as HPLC–DAD samples; residues were completely dried in a 40 °C oven. The total weight of the two parts of dry roots were the dry weight.

### Reagents and chemicals

All solvents were purchased from Merck (Darmstadt, Germany) and were of analytical grade or high-performance liquid chromatography (HPLC) grade. 1,1-Diphenyl-2-picrylhydrazyl (DPPH) and (3-(2-pyridyl)-5,6,diphenyl-1,2,4-triazine-p, p′-disulfonic acid monosodium salt hydrate were from Merck, and Fe^2+^ chloride tetrahydrate ferrozine, and furanocoumarin compound standards, including xanthotoxin, bergapten, oxypeucedanin, imperatorin, phellopterin and isoimperatorin were from Merck. The standards for HPLC with diode-array detection (HPLC–DAD) were dissolved in methanol to a concentration of 100 μg/ml and were stored at − 20 °C.

### Root extracts and sample processing

Freeze-dried root slices were crushed to a power by using a high-speed disintegrator (Solar Energy Co, Taiwan). Extraction for HPLC–DAD was performed as described (Shiau et al. 2011) with some modifications. Methanol (20 ml) was added to 0.2 g ground powder, vortexed and let stand for 30 min. The powder with methanol was then ultrasonic shocked (DC600H, Delta, Taiwan) for 30 min, then centrifuged at 3320 rcf for 3 min. The supernatant was collected and the residue was re-extracted under same conditions for complete extraction. Finally, the supernatants were mixed and filtered through a 0.2-µm Acrodisc Syringe Filter (Pall, USA) before being collected in the Automatic Sampler-approved certified vials (Agilent, USA). Antioxidant assay extraction was performed as described (Shimada et al. 1992) with some modifications. Methanol (20 ml) was added to 1.0 g ground powder; vortexed and shaken overnight (50 rpm), then centrifuged at 3320 rfc for 3 min. The supernatant was collected as the sample.

### HPLC–DAD analysis

To identify the furanocoumarin compounds in root extracts, 25 µl samples were separated by using an analytical column (Syncronis C18, 250 × 4.6 mm, 5 µm particle; ThermoFisher, USA) with flow rate 1.0 ml/min at 30 °C and absorbance 310 nm monitored by the ThermoFisher LC system (ThermoFisher, USA) equipped with a ThermoFinnigan UV detector and ThermoFisher Automatic Sampler. The column was protected by a C18 Guard column (HI-5C18-10C5, Hichrom, UK). A binary gradient was used for H_2_O (Solvent A) and methanol (Solvent B). Optimized pigment separation was achieved with a linear gradient of 40–20% Solvent A for 0–5 min, 20–10% Solvent A for 5–7 min, and 10–0% Solvent A for 7–9 min.

### DPPH assay

DPPH radical scavenging activity was as described (Shimada et al. 1992) with some modifications. The DPPH solution in methanol (0.4 mM) was prepared daily, and antioxidant samples were diluted or concentrated to at least seven concentrations. An amount of 50 µl sample solutions was loaded on a 96-well plate. Each concentration was loaded in six wells. Three were mixed thoroughly with 150 µl fresh DPPH solution (A1) and the others were mixed thoroughly with 150 µl methanol (A2). Pure methanol was used as the control (C1, C2). The loaded 96-well plates were incubated for 30 min in the dark, then the decrease in absorbance of light at 517 nm was measured. The DPPH radical scavenging effects were calculated as [1 − (A1_517_ − A2_517_)/(C1_517_ − C2_517_)].

### Fe^2+^ chelating assay

Fe^2+^ chelating activity was measured as described (Dinis et al. 1994) with some modifications. Antioxidant samples were diluted or concentrated to at least seven concentrations. The Fe^2+^ chloride tetrahydrate solution in methanol (12 mM) was prepared, and 10.5 µl of this solution was mixed thoroughly with 1400 µl sample solution. Ferrozine solution in methanol (5 mM) was prepared. An amount of 200 µl sample solution was loaded on 96-well plates, with each concentration loaded in six wells. Three were thoroughly mixed with 20 µl ferrozine solution (A1) and the others were thoroughly mixed with 150 µl methanol (A2). Pure methanol was used as the control (C1, C2). The loaded 96-well plates were left to stand for 5 min, then the decrease in absorbance of light at 562 nm was measured. The Fe^2+^ chelating effects were calculated as [1 − (A1_562_ − A2_562_)/(C1_562_ − C2_562_)].

### Statistical analysis

Data were analysed by using ANOVA. Source of variation was harvest stage. Significant differences by harvest stage were calculated by the Fisher least significant difference (LSD) tests (*p* < 0.05). Data are expressed as mean ± SD. The IC50 value of DPPH radical scavenging activity and Fe^2+^ chelating activity was predicted by Loess regression. All analyses involved use of R (version 3.2.2) software. p < 0.05 was considered statistically significant.

## Results

### Root weight, length, diameter and moisture content

The transition of Baizhi from vegetative to bolting or summer dormancy, and subsequent root mass is determined by sowing dates (Xingfu et al. 1999), fertilizing management (Derong et al. 1999) and environmental factors such as latitude. Root length and diameter were lower at V-stage than the other two stages, with no significant (*p* < 0.05) difference between B- and S-stage. In Chinese medicine market, lateral roots of ginseng were commercialized independently, which termed as ginseng “fibers” or “beards”. To this, root samples were divided into ‘Taproot’ and ‘Lateral root’ as shown in Table 1. The root lengths were 23.91 ± 1.97, 29.60 ± 5.46 and 30.40 ± 5.83 cm at V-, B- and S-stage, respectively, and the root diameters were 76.45 ± 17.92, 106.93 ± 20.08 and 106.87 ± 38.41 cm, respectively. Root dry weight was highest at S-stage and lowest at B-stage. For taproots, the DW was 2.4 times greater at S- than V-stage and was about 2.7 times greater than at B-stage. For lateral roots, the DW was about 2.2 times greater at S- than V-stage and about 6.8 times greater than at B-stage. V-stage had the highest lateral root to total root ratio, and the proportions of lateral roots were about 26, 11 and 25% at V-, B- and S-stage, respectively. The findings could be explained by a portion of the biomass contributing to the growth of bolting, which may lead to about 2 m of flower moss. In contrast, the above-ground portions die during S-stage. Accordingly, harvesting Baizhi at S-stage rather than at V- or B-stage would allow for better yield. The variation in shoot may affect the harvest weight of root.

**Table 1:** Weight, length, diameter and moisture content of Baizhi roots at three growth stages

	V-stage	B-stage	S-stage
Taproot weight (g)	171.45 ± 99.53^b^*	153.27 ± 37.89^b^	406.96 ± 188.43^a^
Lateral root weight (g)	61.12 ± 35.42^b^	19.66 ± 13.08^c^	133.19 ± 85.02^a^
Total root weight (g)	232.57 ± 128.05^b^	172.93 ± 38.95^c^	540.16 ± 258.82^a^
Lateral root/total root (%)	26	11	25
Root length (cm)	23.91 ± 1.97^b^	29.60 ± 5.46^a^	30.40 ± 5.83^a^
Root diameter (mm)	76.45 ± 17.92^b^	106.93 ± 20.08^a^	106.87 ± 38.41^a^

Data are mean ± SD of 10 independent plants

V-stage, vegetative stage; B-stage, bolting stage; S-stage, summer dormancy stage

* Different letters indicate statistical significance (p < 0.05) by LSD test

### Antioxidant activity

**Fig. 2:**
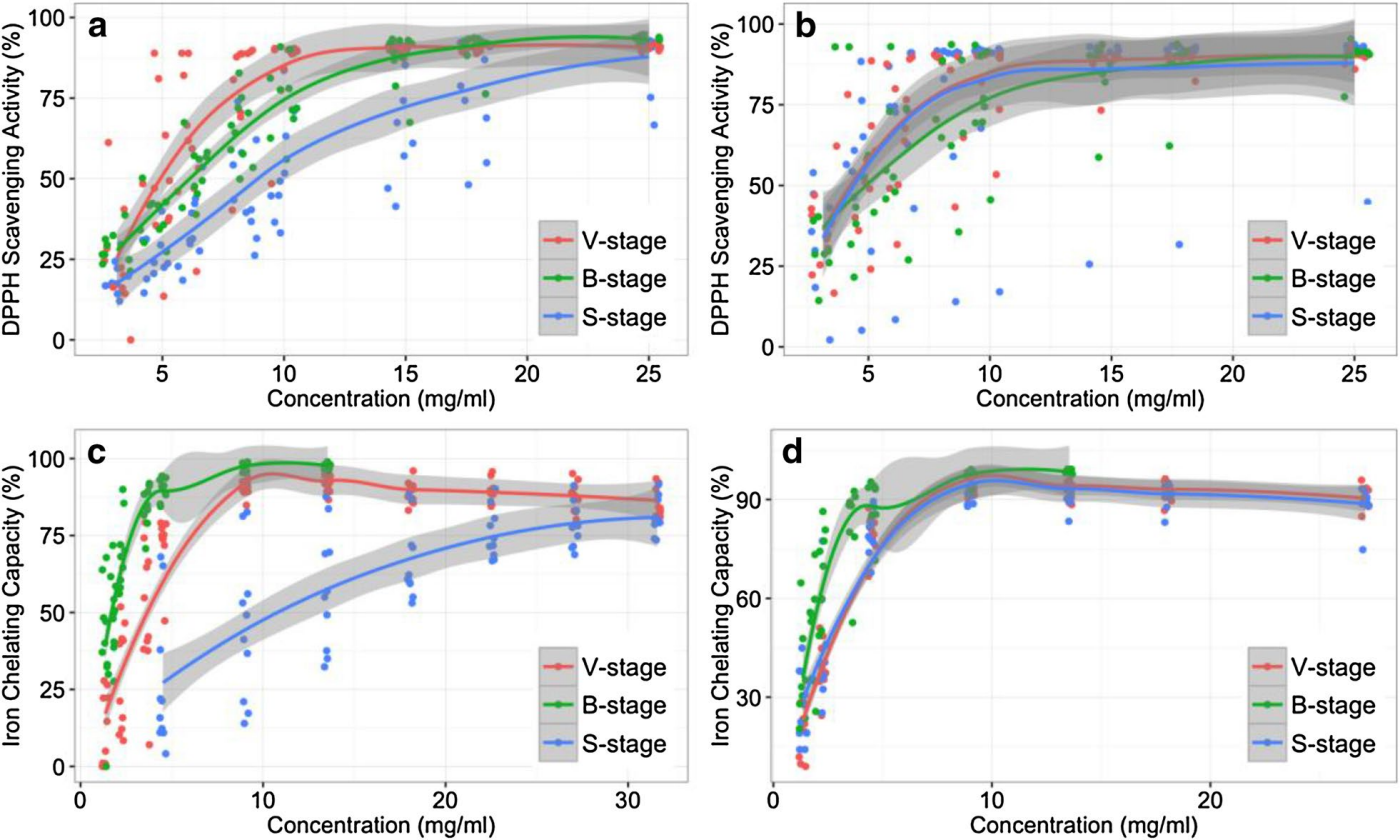
Antioxidant activities of Baizhi root extracts from different growth stages. Baizhi roots were extracted with methanol, and extracts were diluted or concentrated to at least seven concentrations, and subjected to DPPH assay of taproot (**a**) and lateral root (**b**) and Fe^2+^ chelating assay of taproot (**c**) and lateral root (**d**). The gray-line zones indicate 95% confidence intervals for the loess regression line derived from ten plant samples for each concentration

Extracts of Baizhi root have anti-oxidant properties (Pervin et al. 2014; Wang et al. 2017). Both reports have successfully used BHT as a standard to determine the antioxidant activity. Accordingly, in our study, we focused on the content of functional compounds in different harvest stages. We determined DPPH scavenging activity and Fe^2+^ chelating assay by abundant samples with statistical results without the usage of BHT. First, we determined the antioxidant activity of the three stages by DPPH assay and observed a dose-dependent relation in DPPH scavenging activity of Baizhi root extracts (Fig. 2a, b). For taproots, the IC50 value of the extracts were 5.70 ± 4.54, 6.46 ± 3.06 and 10.92 ± 3.82 mg/ml at V-, B- and S-stage, respectively (Table 2). The activity was about 2 times less at S-stage than the other two stages. However, from Table 1, for taproot, the DW was about 2.5 times greater for S-stage than the other stages. The low activity for S-stage may be due to its high DW, which mostly consists of storage compounds such as starch. For extracts of lateral root, the three stages did not differ in activities. The IC50 value of the lateral root extracts were 5.07 ± 5.20, 6.76 ± 5.37 and 6.12 ± 5.12 mg/ml at V-, B- and S-stage, respectively (Table 2). Furthermore, the DW of lateral roots was about 12 times greater at S-stage than the other stages, which indicates that 12 times total activity could be obtained from S-stage than the other stages. Taken together, the highest DPPH radical scavenging activity was obtained from roots harvested at S-stage.

**Table 2:** Antioxidant activity of Baizhi taproot and lateral root at three growth stages

Harvest stage	Taproot	Lateral root
DPPH radical scavenging capacity		
V-stage	5.70 ± 4.54^a^*	5.07 ± 5.20^a^
B-stage	6.46 ± 3.06^a^	6.76 ± 5.37^a^
S-stage	10.92 ± 3.82^b^	6.12 ± 5.12^a^
Fe^2+^ chelating capacity		
V-stage	2.52 ± 7.13^a^	2.55 ± 6.13^a^
B-stage	1.66 ± 1.70^a^	1.90 ± 1.74^a^
S-stage	11.94 ± 6.09^b^	2.65 ± 6.33^a^

Roots were extracted with methanol and subjected to DPPH and Fe^2+^ chelating assay. Activity of extracts is shown as mean ± SD IC50 (mg/ml) predicted by Loess regression

* Different letters indicate statistical significance (p < 0.05) by *t* test

We also determined the antioxidant activity of roots by Fe^2+^ chelating assay and found a dose-dependent relation in Fe^2+^ chelating assay of Baizhi root extracts (Fig. 2c, d). For taproots, the IC50 value of the extracts were 2.52 ± 7.13, 1.66 ± 1.70 and 11.94 ± 6.09 mg/ml for V-, B- and S-stage, respectively (Table 2). The activity was about 5 times less at S-stage than the other two stages (Table 2). Activity in lateral roots did not differ among the stages. The IC50 value for lateral root extracts were 2.55 ± 6.13, 1.90 ± 1.74 and 2.65 ± 6.33 mg/m for V-, B- and S-stage, respectively (Table 2). However, the DW was about 2.5 times greater at S-stage than the other stages. Hence, the active compounds for DPPH and Fe^2+^ chelating mechanisms differed. Baizhi root harvested at S-stage provided antioxidant activity primarily via a DPPH radical scavenging instead of Fe^2+^ chelating mechanism.

### Characterization of six furanocoumarin compounds in Baizhi root

In traditional Chinese medicine, crude Baizhi root materials are used as medicine or functional food for antipyretic and analgesic activities because of abundant furanocoumarin compounds (Li et al. 2014a, b). For example, isoimperatorin, imperatorin and oxypeucedanin may be useful for treating Alzheimer disease (Marumoto and Miyazawa 2010). By analyzing the inhibitory activity on LPS-induced PGE2 production, it has been demonstrated that Isoimperatorin, imperatorin and phellopterin have potential as anti-inflammatory drugs (Ban et al. 2003; Deng et al. 2015). For other furanocoumarin compounds, xanthotoxin, isoimperatorin, imperatorin and oxypeucedanin have anti-cancer abilities (Kim et al. 2007).

**Fig. 3:**
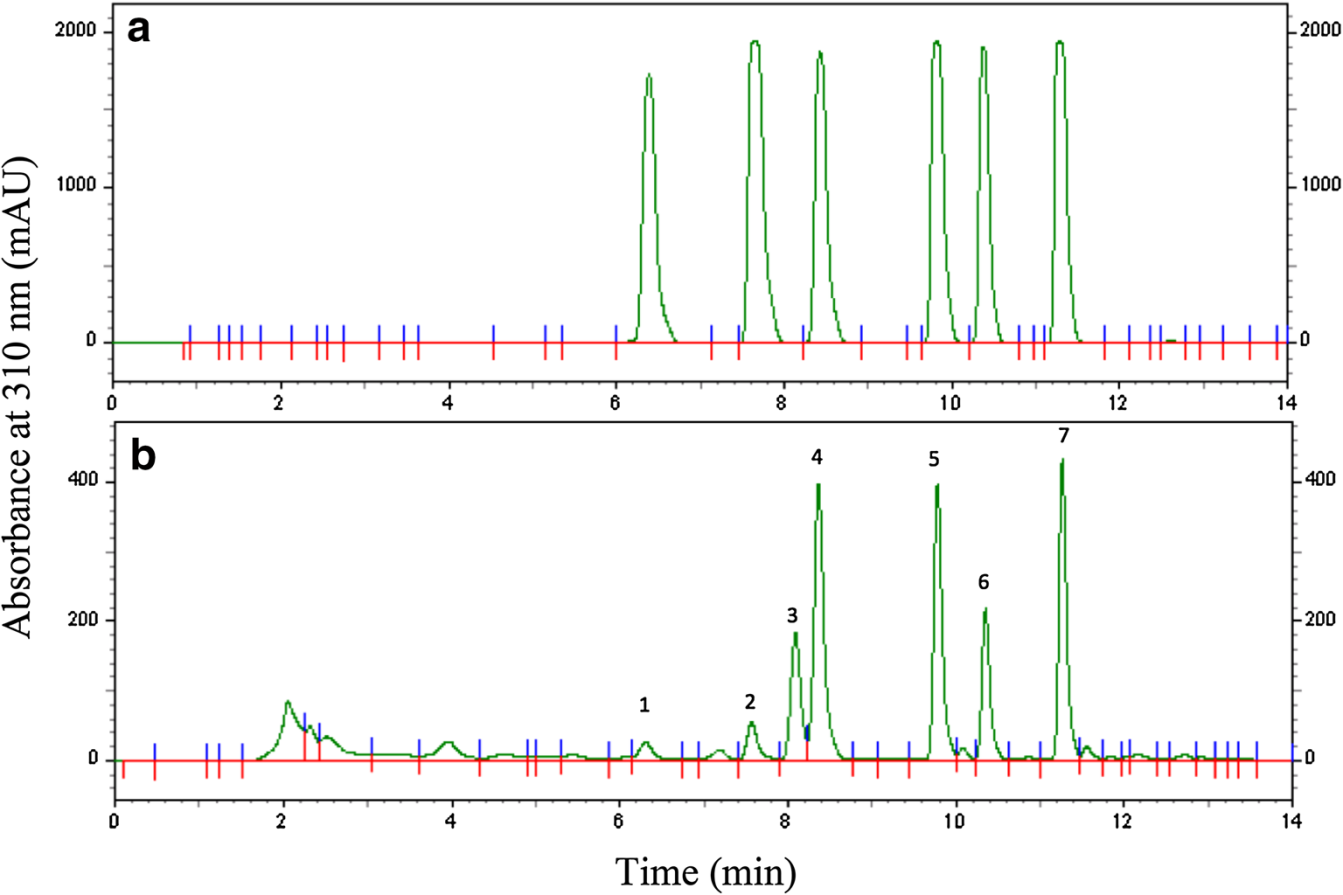
HPLC-DAD chromatogram of standard (**a**) compared with Baizhi root tissue (**b**). Peaks 1, 2, 4, 5, 6 and 7 were identified as xanthotoxin, bergapten, oxypeucedanin, imperatorin, phellopterin and isoimperatorin, respectively

**Fig. 4:**
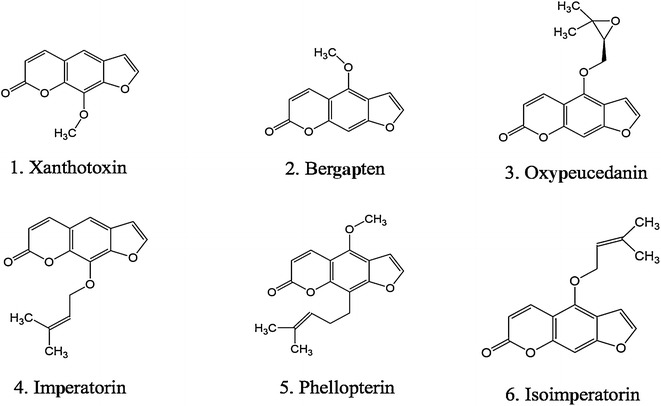
Chemical structure of furanocoumarins identified from root of Baizhi

**Fig. 5:**
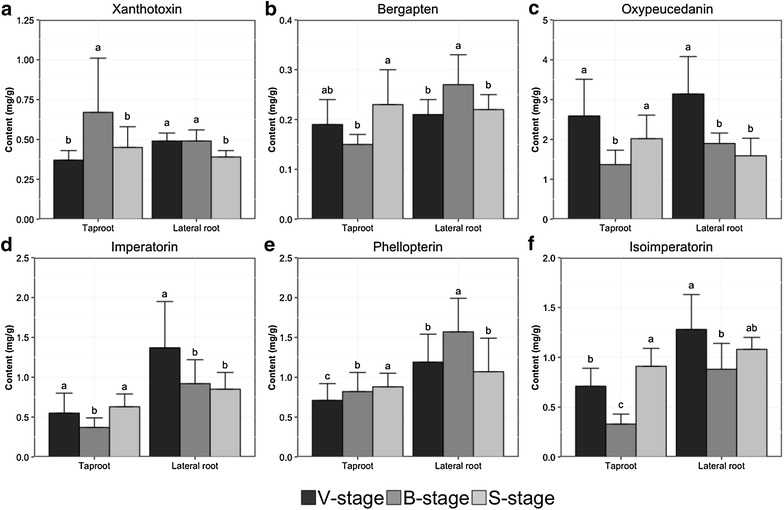
Xanthotoxin (**a**), bergapten (**b**), oxypeucedanin (**c**), imperatorin (**d**), phelllopterin (**e**) and isoimperatorin (**f**) content in Baizhi taproot and lateral root harvested at three stages. Roots were extracted with methanol and subjected to HPLC-DAD analysis (*n *= 10). Data are mean ± SD furanocoumarin content (mg/g sample). Different letters indicate statistical significance (p < 0.05) by LSD test

The chemicals described above involve major furanocoumarin compounds, which we extracted from Baizhi root for HPLC–DAD chromatogram assay; seven clear peaks were obtained. By using reference compounds, we identified peaks 1, 2, 4, 5, 6 and 7 as xanthotoxin, bergapten, oxypeucedanin, imperatorin, phellopterin and isoimperatorin, respectively (Fig. 3) and the chemical structures are in Fig. 4. Then, we analyzed these six furanocoumarins in Baizhi root and found different patterns of furanocoumarin compounds from each harvest stage. The furanocoumarin content in taproot (or lateral root) at each harvest stage showed different patterns (Fig. 5). Oxypeucedanin had the highest yield of the six furanocoumarins (1.5–3.0 mg/g) and the other five yielded from 0.15 mg/g (bergapten) to 1.6 mg/g (phellopterin). The yield of each compound varied less than 50% at different harvest stages except for isoimperatorin from taproot. Similarly, the yield of taproot and lateral root varied less than 50% except for imperatorin and isoimperatorin at B-stage. Additionally, we have also analyzed the furanocoumarin content in shoot (above ground part) of V stage, only imperatorin was detected as about 0.21 mg/g.

**Fig. 6:**
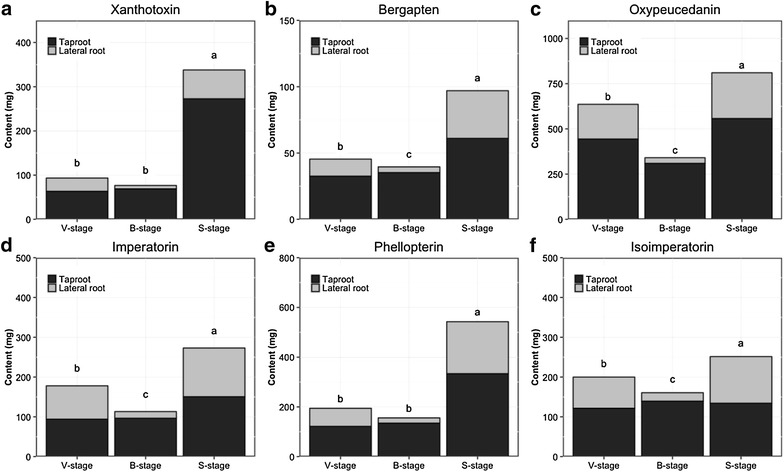
Total content of xanthotoxin (**a**), bergapten (**b**), oxypeucedanin (**c**), imperatorin (**d**), phellopterin (**e**), phellopterin (**f**) in Baizhi taproots and lateral root harvest at three stages. Data are mean of the DW shown at Table 1 multiplied the content of each furanocoumarin at each stage’s taproot (black bars) and lateral root (gray bars). Different letters indicate statistical significance (p < 0.05) by LSD test

To analyze the total amount of the six furanocoumarins in Baizhi roots, the results of harvest yield and six furanocoumarin contents were integrated (Fig. 6). Total furanocoumarin content was highest at S-stage. Also, furanocoumarin content was generally higher at S-stage than the other two stages; especially, the yield of xanthotoxin, bergapten and phellopterin was 2 times higher at S-stage than the other 2 stages. This result strongly supports that S-stage is the best harvest stage than the other stages because of its richer total furanocoumarin content.

## Discussion

The root of Baizhi has been used as a functional food and traditional medicine. In this study, we analyzed the antioxidant activity and total furanocoumarin content of Baizhi root and concluded that the summer dormancy stage is the best stage for harvesting.

Furanocoumarins are believed to function as phytoalexin (Beier and Oertli 1983) or allelochemical compound (Baskin et al. 1967). These compounds are relevant for being potential substitutes for pesticides in crop protection (Eljarrat and Barceló 2001). In wild parsnip (*Pastinaca sativa*, also an Apiaceae plant), the quantity of furanocoumarins allocated to floral units at different stages of development is consistent with the value of those floral units (Nitao and Zangerl 1987). Additionally, studies of field-grown plants showed that FCs storage is closely related to organs and phenological stages. Leaves, stems, roots and fruits do not have equal concentrations of FCs even if psoralen, xanthotoxin, bergapten and isopimpinellin were detected in all tissues (Milesi et al. 2001). Therefore, it is believed that FCs act, specifically in storage organs, as protection chemicals against biostress. As for the case of Baizhi entering the S-stage, the above ground parts occur yellowing, compounds redistribution. FCs are then yielded and accumulated in storage organ (root), specifically in cortex. We suggest that there may be two strategies for Baizhi to survive the stress of summer season. The first strategy is the avoidance trait, in which Baizhi will bolt early in May and adapt the summer season with seed dormancy. The second strategy is the resistance trait, the above-ground parts of Baizhi will senescence and the root will become the resting organs which contain higher dry weigh.

## Conclusions

The results indicate that Baizhi roots should be harvested at S-stage. However, it is possible that the growth of Baizhi switch from vegetative stage to bolting stage directly, without a summer dormancy stage. Our preliminary studies indicated that sowing earlier by 1 month (in November) may result in early bolting in May. Many studies have reported that stage-transition of Baizhi is determined by, for example, sowing date, fertilizing management and other environmental factors. In the near future, global warming is expected to affect phenological events such as flowering and fruiting in plants and will hasten development time in plant species that respond to cues such as degree days (Hughes 2000). Under the threat of global warming, analyzing the stage-transition physiology of Baizhi will facilitate the modification of the cropping system to achieve a stable and uniform S-stage of Baizhi.

## Abbreviations

V-stage: vegetative stage
S-stage: summer dormancy stage
B-stage: bolting stage
DW: dry weight
HPLC–DAD: high-performance liquid chromatography with diode-array detection
DPPH: 2,2-diphenyl-l-picrylhydrazyl
